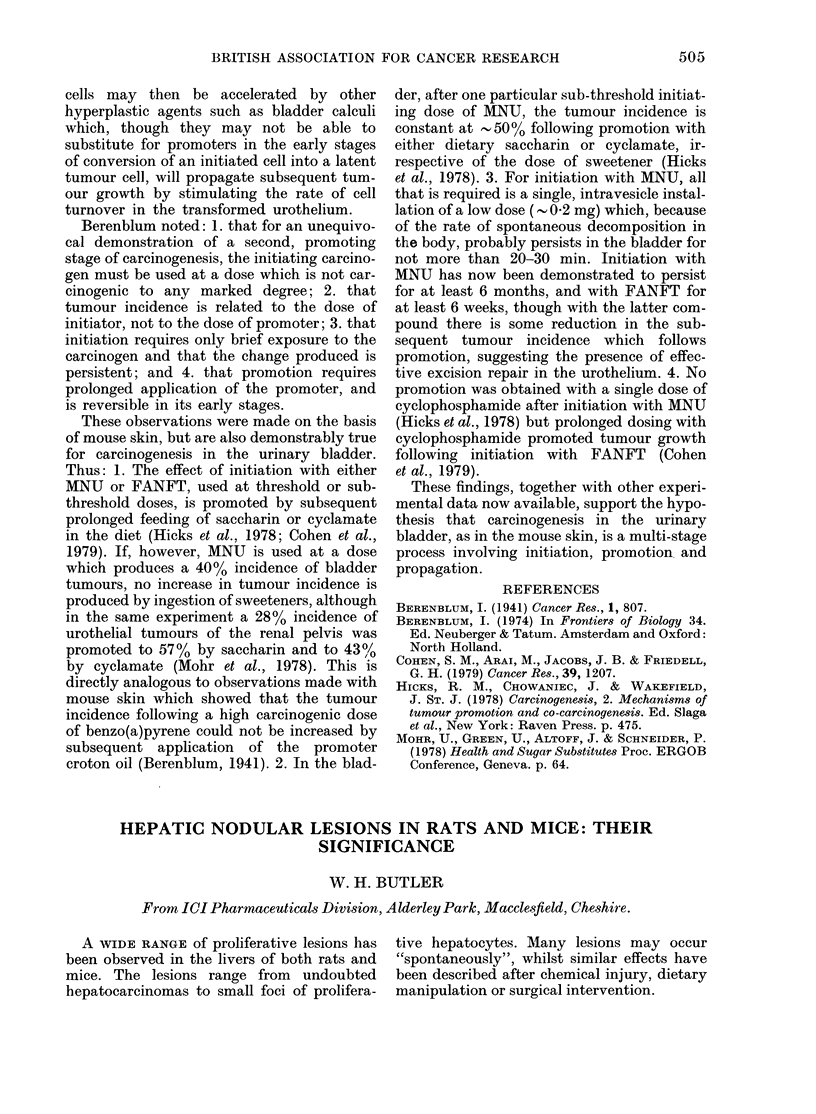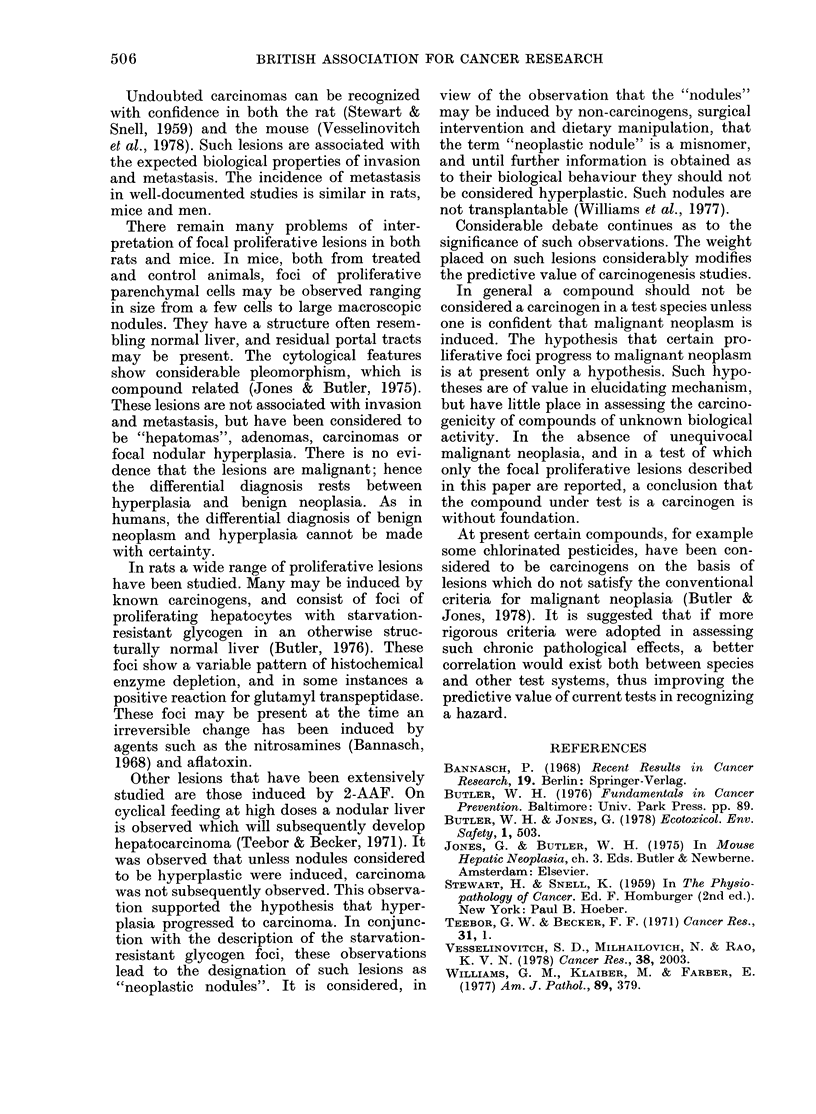# Hepatic nodular lesions in rats and mice: their significance.

**DOI:** 10.1038/bjc.1980.85

**Published:** 1980-03

**Authors:** W. H. Butler


### HEPATIC NODULAR LESIONS IN RATS AND MICE: THEIR SIGNIFICANCE

W. H. BUTLER

*From ICI Pharmaceuticals Division, Alderley Park, Macclesfield, Cheshire.*

A wide range of proliferative lesions has been observed in the livers of both rats and mice. The lesions range from undoubted hepatocarcinomas to small foci of proliferative hepatocytes. Many lesions may occur "spontaneously", whilst similar effects have been described after chemical injury, dietary manipulation or surgical intervention.

Undoubted carcinomas can be recognized with confidence in both the rat (Stewart & Snell, 1959) and the mouse (Vesselinovitch *et al.*, 1978). Such lesions are associated with the expected biological properties of invasion and metastasis. The incidence of metastasis in well-documented studies is similar in rats, mice and men.

There remain many problems of interpretation of focal proliferative lesions in both rats and mice. In mice, both from treated and control animals, foci of proliferative parenchymal cells may be observed ranging in size from a few cells to large macroscopic nodules. They have a structure often resembling normal liver, and residual portal tracts may be present. The cytological features show considerable pleomorphism, which is compound related (Jones & Butler, 1975). These lesions are not associated with invasion and metastasis, but have been considered to be "hepatomas", adenomas, carcinomas or focal nodular hyperplasia. There is no evidence that the lesions are malignant; hence the differential diagnosis rests between hyperplasia and benign neoplasia. As in humans, the differential diagnosis of benign neoplasm and hyperplasia cannot be made with certainty.

In rats a wide range of proliferative lesions have been studied. Many may be induced by known carcinogens, and consist of foci of proliferating hepatocytes with starvation-resistant glycogen in an otherwise structurally normal liver (Butler, 1976). These foci show a variable pattern of histochemical enzyme depletion, and in some instances a positive reaction for glutamyl transpeptidase. These foci may be present at the time an irreversible change has been induced by agents such as the nitrosamines (Bannasch, 1968) and aflatoxin.

Other lesions that have been extensively studied are those induced by 2-AAF. On cyclical feeding at high doses a nodular liver is observed which will subsequently develop hepatocarcinoma (Teebor & Becker, 1971). It was observed that unless nodules considered to be hyperplastic were induced, carcinoma was not subsequently observed. This observation supported the hypothesis that hyperplasia progressed to carcinoma. In conjunction with the description of the starvation-resistant glycogen foci, these observations lead to the designation of such lesions as "neoplastic nodules". It is considered, in view of the observation that the "nodules" may be induced by non-carcinogens, surgical intervention and dietary manipulation, that the term "neoplastic nodule" is a misnomer, and until further information is obtained as to their biological behaviour they should not be considered hyperplastic. Such nodules are not transplantable (Williams *et al.*, 1977).

Considerable debate continues as to the significance of such observations. The weight placed on such lesions considerably modifies the predictive value of carcinogenesis studies.

In general a compound should not be considered a carcinogen in a test species unless one is confident that malignant neoplasm is induced. The hypothesis that certain proliferative foci progress to malignant neoplasm is at present only a hypothesis. Such hypotheses are of value in elucidating mechanism, but have little place in assessing the carcinogenicity of compounds of unknown biological activity. In the absence of unequivocal malignant neoplasia, and in a test of which only the focal proliferative lesions described in this paper are reported, a conclusion that the compound under test is a carcinogen is without foundation.

At present certain compounds, for example some chlorinated pesticides, have been considered to be carcinogens on the basis of lesions which do not satisfy the conventional criteria for malignant neoplasia (Butler & Jones, 1978). It is suggested that if more rigorous criteria were adopted in assessing such chronic pathological effects, a better correlation would exist both between species and other test systems, thus improving the predictive value of current tests in recognizing a hazard.

### REFERENCES

Bannasch, P. (1968) *Recent Results in Cancer Research*, **19.** Berlin: Springer-Verlag.

Butler, W. H. (1976) *Fundamentals in Cancer Prevention.* Baltimore: Univ. Park Press. pp. 89.

Butler, W. H. & Jones, G. (1978) *Ecotoxicol. Env. Safety*, **1**, 503.

Jones, G. & Butler, W. H. (1975) In *Mouse Hepatic Neoplasia*, ch. 3. Eds. Butler & Newberne. Amsterdam: Elsevier.

Stewart, H. & Snell, K. (1959) In *The Physiopathology of Cancer.* Ed. F. Homburger (2nd ed.). New York: Paul B. Hoeber.

Teebor, G. W. & Becker, F. F. (1971) *Cancer Res.*, **31**, 1.

Vesselinovitch, S. D., Milhailovich, N. & Rao, K. V. N. (1978) *Cancer Res.*, **38**, 2003.

Williams, G. M., Klaiber, M. & Farber, E. (1977) *Am. J. Pathol.*, **89**, 379.